# Splice-Variant Knock-Out of TGFβ Receptors Perturbates the Proteome of Ovarian Carcinoma Cells

**DOI:** 10.3390/ijms222312647

**Published:** 2021-11-23

**Authors:** Liora Jacobs Catane, Ofra Moshel, Yoav Smith, Ben Davidson, Reuven Reich

**Affiliations:** 1 Institute of Drug Research, School of Pharmacy, Faculty of Medicine, The Hebrew University-Hadassah Medical School, Jerusalem 91120, Israel; liora.jacobs@mail.huji.ac.il; 2 Core Research Facility, Institute for Drug Research, School of Pharmacy, Faculty of Medicine, The Hebrew University-Hadassah Medical School, Jerusalem 91120, Israel; oframo@savion.huji.ac.il; 3 Genomic Data Analysis Unit, The Hebrew University of Jerusalem-Hadassah Medical School, P.O. Box 12272, Jerusalem 91120, Israel; yoavs@ekmd.huji.ac.il; 4 Department of Pathology, Oslo University Hospital, Norwegian Radium Hospital, N-0310 Oslo, Norway; ben.davidson@medisin.uio.no; 5 Faculty of Medicine, Institute of Clinical Medicine, University of Oslo, N-0316 Oslo, Norway

**Keywords:** transforming growth factor-β, TGFβ, receptor splice variants, ovarian carcinoma, proteomics, CRISPR/Cas9 KO’s

## Abstract

The aim of this study was to analyze the biological role of different transforming growth factor-β (TGFβ) receptor splice variants in ovarian carcinoma (OC). Specific receptor variant knockouts (KO) were prepared using the CRISPR/Cas9 genome editing system in two OC cell lines, TβRI variant 1 (TβRIv1) KO in ES-2 cells and TβRII variant 1 (TβRIIv1) KO in OVCAR-8 cells. Control and KO cells were compared by proteomic analysis, functional tests, analysis of epithelial–mesenchymal transition (EMT) drivers, and Western blot of signaling proteins. Proteomic analysis revealed significant changes in protein pathways in the KO cells. TβRIv1 KO resulted in a significant reduction in both cellular motility and invasion, while TβRIIv1 KO significantly reduced cellular motility and increased Reactive Oxygen Species (ROS) production. Both receptor variant KOs reduced MET protein levels. Of the EMT drivers, a significant decrease in TWIST protein expression, and increase in SNAIL protein and *MALAT1* mRNA levels were observed in the TβRIIv1 KO compared to control. A significant decrease in JNK1 and JNK2 activation was found in the TβRIv1 KO compared to control cells. These findings provide new insight regarding the biological role of the TGFβ receptor variants in the biology and potentially the progression of OC.

## 1. Introduction

Transforming growth factor β is a superfamily of cytokines consisting of several structurally related polypeptide growth factors including TGFβs, activins, inhibins, and others. TGFβ is involved in many different cellular processes, such as cell proliferation, differentiation, motility, adhesion, embryogenesis, fibrosis, angiogenesis, immunosuppression, and apoptosis [1,2]. TGFβ has a dual role and can act both as tumor suppresser and tumor promoter, a phenomenon known as the “TGFβ paradox” [3,4]. In early stages of disease it can prevent cell growth and promote apoptosis, thus acting as tumor suppressor; yet, in later stages it can cause the cancer to acquire an invasive and metastatic phenotype, thereby acting as a tumor promoter [5].

In mammals, the TGFβ family consists of three ligands, TGFβ_1–3_, and three transmembrane receptors, TβRI–III. The type I and type II receptors are serine threonine kinase receptors. The type III receptor is a membrane-anchored proteoglycan, betaglycan, and acts as a co-receptor [6]. Structural studies have shown that a dimeric ligand molecule binds the type ΙΙ receptor, which stimulates the generation of a stabilized hetero-hexameric complex containing two ligands and two type Ι and two type ΙΙ receptors [7].

The second ligand as well as the three receptors undergo alternative splicing to create a number of variants [8]. Other than TβRΙI [9], it is not yet known whether or not all variants undergo translation to active proteins. TβRΙ consists of three known variants to date [10]. Variant 3 differs from variant 1 by a sequence of 12 nucleotides at the beginning of exon 3. Variant 2 is missing exon 3, which includes part of the GS domain [11] that undergoes phosphorylation, which may result in an inactive protein. TβRΙI consists of two variants. Variant 2 is the short transcript variant, missing exon 2, which is part of the N-terminal extracellular domain and includes glycosylation sites [12]. TβRΙII has four variants that differ from the longest transcript in the 5′ UTR as well as in the translated region by only three base pairs [13].

Ovarian carcinoma (OC) is the most lethal gynecologic cancer, mainly owing to diagnosis at advanced stage (FIGO III–IV) and intrinsic or acquired chemoresistance. Malignant effusions are found in many patients at both diagnosis and disease recurrence [14]. TGFβ signaling has been extensively studied in OC [14]. In our previous study we analyzed the mRNA expression and clinical relevance of the TGFβ ligands and receptors and the activation of the canonical pathway in high-grade serous carcinoma (HGSC), which represents ~70% of all OC. TGFβ receptor splice variant expression was anatomic site dependent in HGSC [13]. Our objective for the present study was to elucidate further knowledge on the contribution of the TGFβ pathway to the progression of OC. Furthermore, we aimed to define the biological role of the different receptor variants. Using CRISPR/Cas9 methodology, we analyzed the role of the receptor splice variants on cellular proteome and on metastatic behavior in OC cell lines.

## 2. Results

### 2.1. CRISPR/Cas9 KO in OC Cell Lines

**Figure 1:**
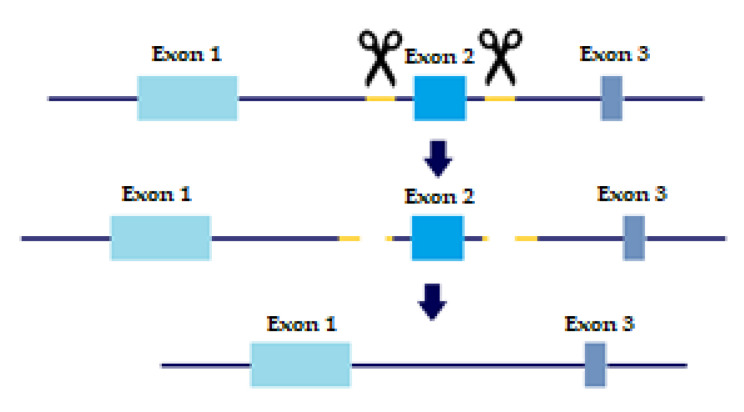
CRISPR sgRNA design. Yellow marks sgRNA, scissors are CAS9 enzyme. Two sgRNA templates are designed to flag both upstream and downstream of the Exon we wish to eliminate. Two double-strand breaks are created and the cell machinery repairs the DNA, excluding the undesired Exon.

In order to evaluate the precise role of TGFβ receptors and their variants in metastatic behavior and tumor progression, KO cell lines were created with a slightly altered protocol of CRISPR/CAS9 based on Wang, H.C. et al. [15]. As mentioned above, TβRI and TβRII each have two distinct variants. *TβRI* variant 2 is missing Exon 3, which is present in variant 1. *TβRII* variant 2 is missing Exon 2, which is present in variant 1. Briefly, sgRNAs were designed to flank each of the exons we wished to delete, thus creating a double-strand break upstream and downstream of the exon. The repair machinery would then ligate the DNA without the exon, thus removing the long variant of the TGFβ receptors from the cell genome (Figure 1).

Plasmid transfection was done in the ES-2 and OVCAR-8 cell lines, and the following percentages of KOs were generated: 60% KO of *TβRI* variant 1 (*TβRIv1*) in ES-2 cell line and 100% KO of *TβRII* variant 1 (*TβRIIv1*) in OVCAR-8 cell line.

**Figure 2:**
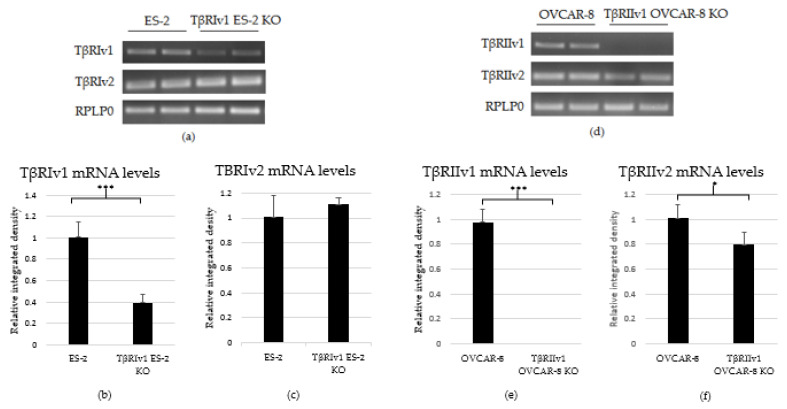
PCR analysis of the gene expression of *TβRI* (variant 1 and variant 2) and *TβRII* (variant 1 and variant 2) in cDNA created from single-cell CRISPR/Cas9 transfected ES-2 and OVCAR-8 cell lines, respectively, as well as their relative control. (**a**–**c**) Agarose gel electrophoresis and quantification of band density by Image-J software showed a 60% reduction in the expression levels of *TβRIv1* in the TβRIv1 ES-2 KO. No significant changes in the expression of *TβRIv2* were seen. (**d**–**f**) Agarose gel electrophoresis and quantification of band density by Image-J software showed complete KO of *TβRIIv1* in the TβRIIv1 OVCAR-8 KO and a significant decrease in the expression levels of *TβRIIv2*. * *p* < 0.05 and *** *p* < 0.001.

As there are no specific antibodies to the receptor splice variants, verification of KO generation was done through examination of the mRNA. RNA was extracted from the cells, followed by cDNA synthesis and PCR. PCR products were then run on 1.5% agarose gel electrophoresis. Exclusion of the whole exon from the DNA would result in no mRNA sequence for the target gene variant. As evident from Figure 2, there was complete KO for *TβRIIv1* in the OVCAR-8 cell line. Band density analysis showed a 60% KO of *TβRIv1* in ES-2 cells. mRNA PCR analysis was performed in order to assess whether or not the cells compensate for the lack of silencing variant 1 of the abovementioned receptor via upregulating variant 2. Surprisingly, TβRIIv1 OVCAR-8 KO cells had significant decrease in the levels of TβRII variant 2 (*TβRIIv2)* compared to control OVCAR-8 cells (Figure 2f). This reduction may reflect the possibility that the receptor variants’ mRNAs might be part of a yet-unknown ceRNA cycle that regulates downstream events in the progression of OC. The ES-2 KO did not show any significant difference in the levels of TβRI variant 2 (*TβRIv2)* (Figure 2c).

### 2.2. KO Formation Results in Significant Changes in the Cellular Proteome

In order to determine the changes in the biological pathways induced by KO formation, proteins were extracted from both KO and control cells and analyzed for changes.

#### 2.2.1. ES-2 vs. TβRIv1-ES-2 KO

**Figure 3:**
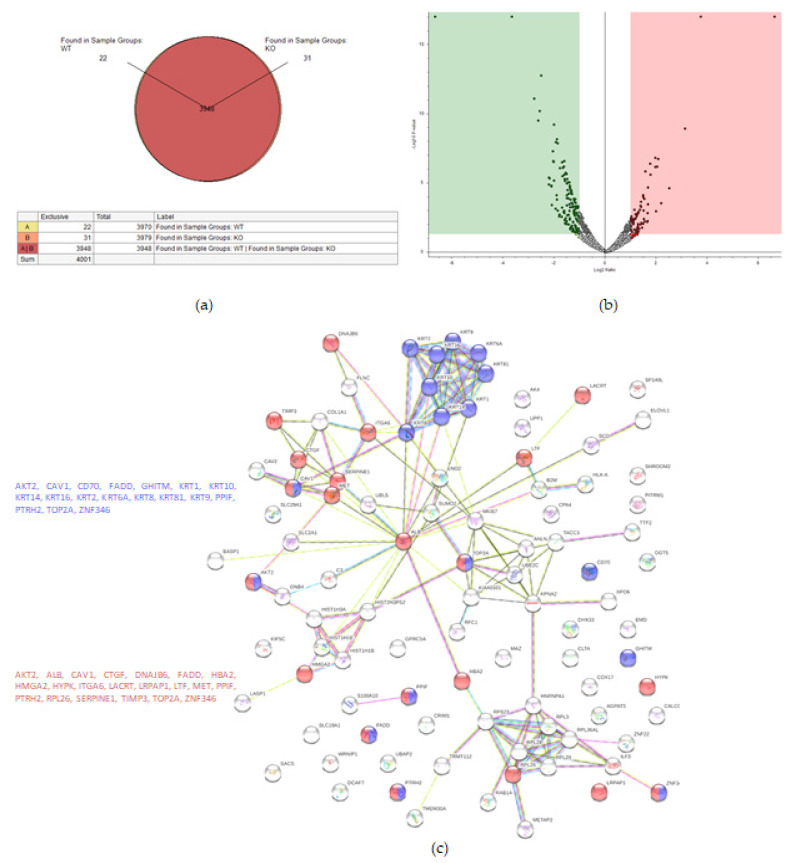
Proteomic analysis of TβRIv1 ES-2 KO and ES-2 control. (**a**) Venn diagram showing 3948 commonly expressed proteins (red), 22 proteins present only in controls (yellow), and 31 proteins found in KO only (orange). (**b**) Volcano plot. Red = proteins significantly upregulated in KO; green = proteins significantly downregulated in KO. (**c**) String protein–protein interactions of significantly downregulated proteins (blue = programmed cell death, red = regulation of cell death).

Proteomic analysis detected 4001 proteins in total, of which 22 and 31 were unique for the WT and KO samples, respectively (Figure 3a). Fifty-eight proteins were upregulated in the KO cells (abundance ratio > 2-fold, *p* < 0.05), and 118 proteins were downregulated in KO cells (abundance ratio < 0.5, *p* < 0.05).

String analysis showed downregulation of several pathways, one of which was a downregulation of proteins concerning cell death (Figure 3c), of which 16 proteins were involved in general regulation of cell death and 17 proteins were involved in negative regulation of cell death.

#### 2.2.2. OVCAR-8 v.s. TβRIIv1 OVCAR-8 KO

**Figure 4:**
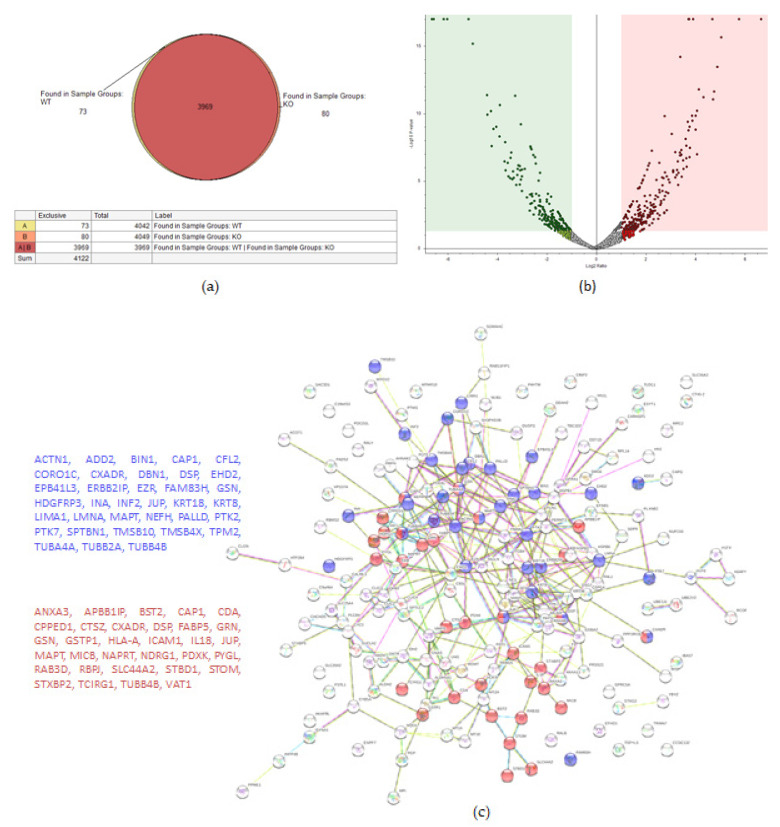

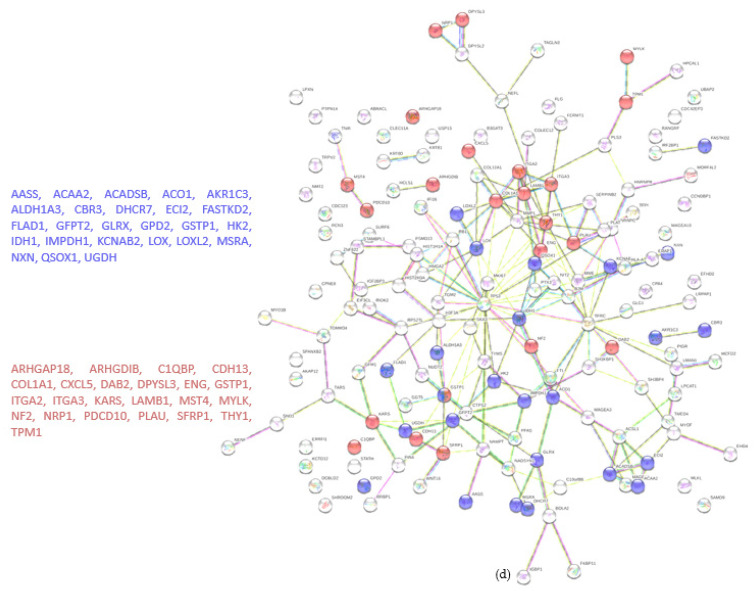
Proteomic analysis of TβRIIv1 OVCAR-8 KO and OVCAR-8 control. (**a**) Venn diagram, 3969 proteins in common (red), 73 proteins in WT only (yellow), and 80 proteins found in KO only (orange). (**b**) Volcano plot, red = proteins significantly upregulated in KO, green = proteins significantly downregulated in KO. (**c**) String protein–protein interactions of significantly upregulated proteins (blue = cytoskeleton organization, red = immune system activation). (**d**) String protein-protein interactions of significantly downregulated proteins (blue = oxidation reduction processes, red = regulation of cell motility).

A total of 4122 proteins were detected, of which 73 and 80 were unique for the WT and KO groups, respectively (Figure 4a). There were 206 proteins found to be upregulated in the KO group compared to the WT (abundance ratio > 2-fold, *p* < 0.05). Among several biological pathways, String analysis showed upregulation of proteins involved in immune system activation and cytoskeletal organization (Figure 4c). There were 175 proteins downregulated (abundance ratio < 0.5, *p* < 0.05). String analysis showed downregulated proteins related to oxidation reduction processes and cell motility (Figure 4d).

### 2.3. Pro-MET and MET Proteins Are Downregulated in the KOs’, and ROS Formation Is Upregulated in the TβRIIv1 OVCAR-8 KO

**Figure 5:**
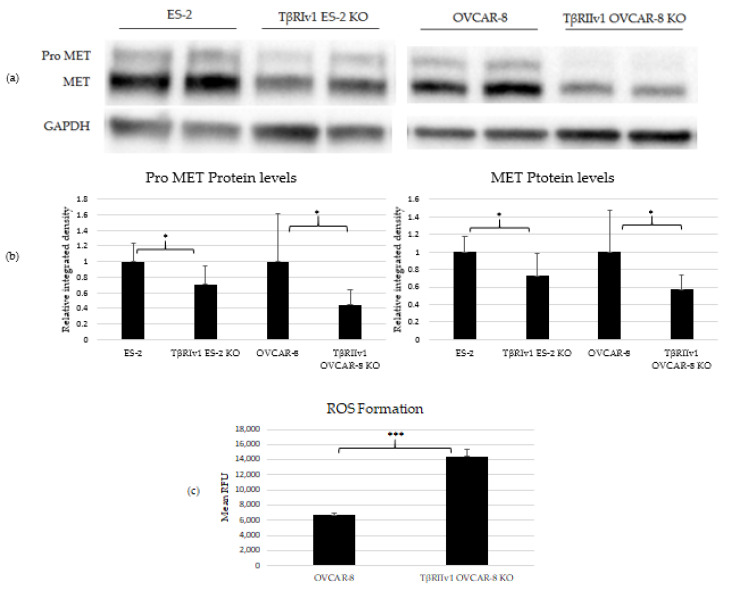
KO formation significantly reduced levels of pro-MET (175 kDa) and MET (145 kDa) in both KOs created and increased ROS’ formation in the TβRIIv1 OVCAR-8 KO. (**a**) Representative Western Blot of Pro-MET and MET for ES-2 control, TβRIv1 ES-2 KO, OVCAR-8, and TβRIIv1 OVCAR-8 KO. (**b**) Western blot quantification for the abovementioned cell lines. Both TβRIv1 ES-2 KO and TβRIIv1 OVCAR-8 KO expressed significantly lower levels of Pro-MET and MET compared to their relative controls. (**c**) Quantification of ROS levels: Mean RFU of the oxidative stress indicator CM-H2DCFDA in OVCAR-8 and TβRIIv1 OVCAR-8 KO cells. KO formation increased ROS levels in cells. (* *p* < 0.05, *** *p* < 0.001).

Three proteins were chosen to validate the proteomic analyses. MET and CAV1 are both negative regulators of cell death, and p53 is also known to be involved in cell death as well as oxidation-reduction processes. Western Blot analyses was done to estimate the protein levels in the cell lysates. Of these three proteins, only MET and its precursor, Pro-MET, showed a significant difference between control and KO cells. Both TβRIv1 ES-2 KO and TβRIIv1 OVCAR-8 KO expressed significantly lower levels of Pro-MET and MET protein levels compared to their relative control (Figure 5). These results corresponded to the downregulation observed in the proteomic analyses. Downregulation of MET, a negative regulator of cell death, may enhance cell death in the KO cells compared to the control.

In order to relate to oxidation-reduction processes and oxidative stress, ROS levels were quantified. Cells were exposed to 1 µM of CM-H_2_DCFDA, a general ROS indicator that reacts with glutathione and other thiols to yield a fluorescent adduct. Fluorescence was measured and quantified using a plate reader fluorometer. TβRIIv1 OVCAR-8 KO showed significantly higher ROS levels compared to OVCAR-8 control cells, exhibiting a downregulation in the regulation of oxidative stress, as seen in the string analysis above.

### 2.4. KO Formation Causes a Decrease in the Migratory and Invasive Capabilities of the Cells

In order to evaluate the cellular functions that were affected as a response of KO formation, we applied several cellular assays and examined the expression of EMT inducers. Proliferation was examined using the MTT assay, Matrix Metalloproteinase (MMP) activity through the zymography assay, and cell death was examined via caspase-3 activity. None of these assays revealed significant changes between the KO and control cells.

**Figure 6:**
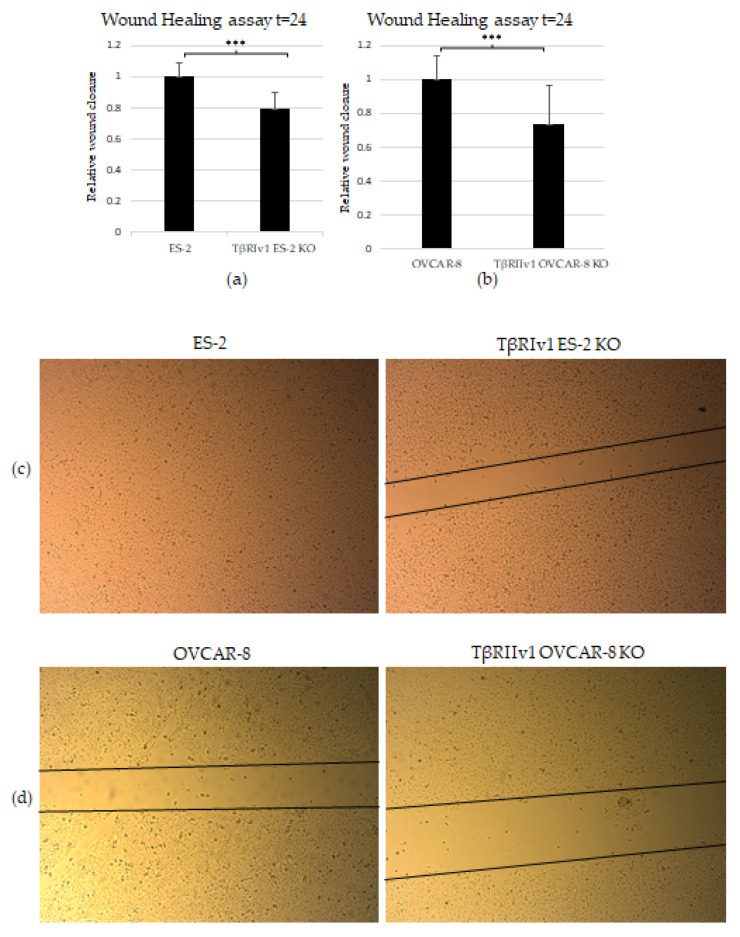

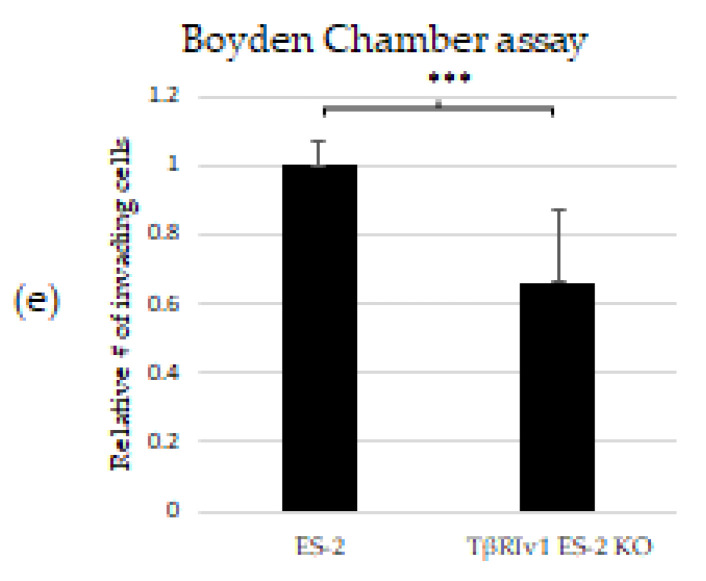
KO formation significantly reduced the migratory and invasive properties of the cells. (**a**,**b**) Quantification of Wound-healing assay at t = 24 h demonstrated that both TβRIv1 ES-2 KO and TβRIIv1 OVCAR-8 KO (respectively) showed a significant decrease in the ability to migrate and close the gap made compared to controls. (**c**,**d**) Representative image of wound-healing assay for TβRIv1 ES-2 KO and TβRIIv1 OVCAR-8 KO and their relative controls (respectively) at t = 24 h. Both KO cell lines migrated slower and showed significantly lower gap closure compared to their relative controls. (**e**) Quantification of Boyden Chamber assay for ES-2 vs. TβRIv1ES-2 KO. KO formation caused a significant decrease in the cells’ ability to cross the Matrigel membrane, showing lower invasive capabilities of the KO cells compared to control cells (*** *p* < 0.001).

The scratch test (wound-healing assay) was applied to examine the contribution of the TGFβ receptor splice variants on the migratory capabilities of the cells. Both TβRIv1 ES-2 KO and TβRIIv1 OVCAR-8 KO exhibited slower wound closure, migrating significantly less than their relevant control at t = 24 h (Figure 6a,b). This observation can relate as well to the proteomic results showing a downregulation in proteins involved in regulation of cell motility in the TβRIIv1 OVCAR-8 KO.

In order to examine the effect of the splice variants on the invasive properties of the cells, we applied the Matrigel Invasion (Boyden Chamber) assay. TβRIv1 ES-2 KO showed a lower number of infiltrating cells across the Matrigel-coated membrane, exhibiting significantly less invasion than ES-2 control cells (Figure 6e). TβRIIv1 OVCAR-8 KO did not show significant invasive differences compared to control OVCAR-8 cells.

### 2.5. EMT Induction Is Splice-Variant Dependent

**Figure 7:**
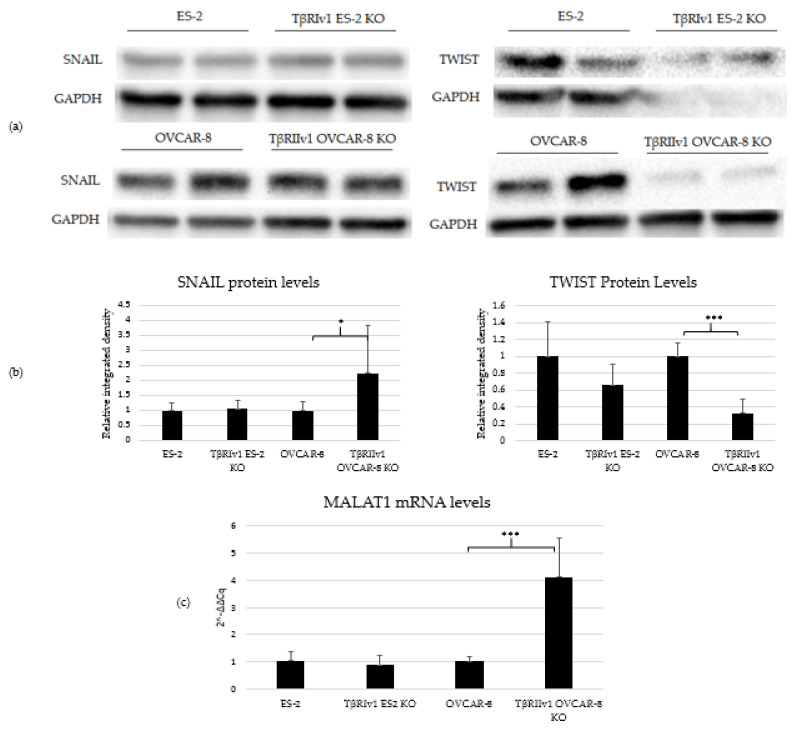
TβRIIv1 OVCAR-8 KO expressed significantly higher protein levels of SNAIL (29 kDa) and *MALAT1* mRNA, yet significantly lower protein levels of TWIST (26 kDa) compared to the OVCAR-8 control cells. (**a**) Representative Western Blot of SNAIL and TWIST for ES-2 control, TβRIv1 ES-2 KO, OVCAR-8, and TβRIIv1 OVCAR-8 KO. (**b**) Western blot quantification for the abovementioned cell lines and proteins. TβRIv1 ES-2 KO expressed similar SNAIL protein levels and expressed marginally lower (*p* value= 0.06) TWIST protein levels compared to ES-2 control. TβRIIv1 OVCAR-8 KO expressed significantly higher protein levels of SNAIL, yet significantly lower protein levels of TWIST compared to OVCAR-8 control cells. (**c**) Relative mRNA expression levels of the long, non-coding RNA *MALAT1* in the KO and control cell lines. Results given are relative to control, calculated as 2^−ΔΔCq^. There were no significant differences in mRNA levels of *MALAT1* between the TβRIv1 ES-2 KO compared to control. *MALAT1* mRNA levels were significantly higher in the TβRIIv1 OVCAR-8 KO compared to control. (* *p* < 0.05, *** *p* < 0.001).

TGFβ signaling is involved in EMT in tumor cells, including OC. In order to determine whether receptor variant KO had an effect on EMT initiated by TGFβ, we analyzed the expression of two regulatory elements of this process, SNAIL and TWIST, via Western Blot Analysis. TβRIv1ES-2 KO cells showed a marginal reduction in the expression of TWIST protein compared to ES-2 control cells, while TβRIIv1 OVCAR-8 KO cells showed a significant increase in SNAIL protein levels yet a significant decrease in *TWIST* expression compared to OVCAR-8 control cells (Figure 7a,b).

Previous studies showed that TGFβ2 induces overexpression of EMT markers via a *MALAT1*-dependent mechanism that acts as a ceRNA targeting Smad4 and also binds to phospho-Smad2/3 [16,17,18]. We, therefore, analyzed the expression of *MALAT1* in our KO cells. Deletion of *TβRIIv1* affected the expression of *MALAT1,* while deletion of *TβRIv1* failed to do so (Figure 7c).

### 2.6. KO Formation Affects Downstream Signaling

**Figure 8:**
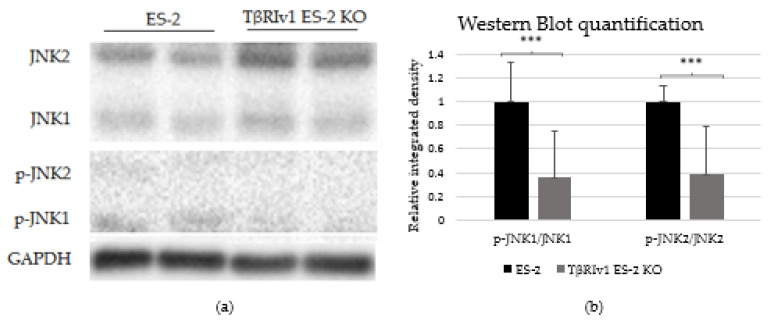
TβRIv1 ES-2 KO showed significantly lower activation of the JNK1/2 non-canonical pathway. (**a**) Representative Western blot assay of JNK1/2 (46 kDa and 54 kDa, respectively) and p-JNK1/2 (46 kDa and 54 kDa, respectively) for TβRIv1 ES-2 KO and ES-2 control cells. (**b**) Quantification of Western blot assay. Results are shown as ratio of phospho-protein/protein. Both JNK1 activation and JNK 2 activation were significantly decreased in TβRIv1 ES-2 KO compared to the ES-2 control. (*** *p* < 0.001).

In our previous study on a large cohort of OC samples, we demonstrated differential expression of TGFβ receptor splice variants in the different anatomical sites of the disease and that effusion-derived tumor cells did not show Smad activation as part of the TGFβ canonical pathway [13]. Further, in another study, using the same sample cohort, we demonstrated activation of several pathways also known to be part of the non-canonical TGFβ signaling pathways [19]. In order to determine whether receptor variants had an effect on the activation/regulation of the non-canonical TGFβ pathways, we correlated the expression of the variants in clinical material with the non-canonical TGFβ pathway signals in the same samples. Western blot analysis was focused on specific proteins chosen according to their clinical significance in patient samples [19]. In clinical specimens, ERK ratio was found to be significantly positively related to *TβRIIv2* (*p* = 0.041), p-AKT levels were higher in post-chemotherapy compared to chemo-naïve samples (*p* = 0.029), and JNK1 levels were higher in effusions from patients with less favorable chemotherapy response at diagnosis compared to those with complete response (*p* = 0.045). Analyzing these three proteins’ activation in the KO-generated cells revealed that only TβRIv1-ES-2 KO presented significant differences compared to its control, ES-2. The p-JNK1/JNK1 ratio was significantly lower in the KO, as was the p-J NK2/JNK2 ratio (Figure 8). AKT and ERK activation showed no significant differences (figures not shown). It is clear from these results that this splice variant was involved in the activation/regulation of the JNK1/2 pathways.

## 3. Discussion

TGFβ inhibits cell growth in benign cells while promoting progression in certain cancers, described as the “TGFβ paradox”. Studies of metastatic OC have, nevertheless, documented mainly a tumor-promoting effect for this pathway to date. OC cells isolated from metastases were more TGFβ-responsive than those isolated from the ovarian tumors [20]. Stromal progenitor cells isolated from OC ascites had high TGFβ expression [21]. TGFβ blockade in a mouse model inhibited OC growth by negatively regulating proliferation and angiogenesis and suppressed ascites formation [22]. It is additionally unknown whether the different splice variants of TGFβ ligands and receptors are translated to proteins. TGFβRII is exceptional, since there are publications showing that its two variants are translated to active proteins that activate the canonical pathway [9] and have different affinity towards different ligands [23]. The precise role of the splice variants of the TGFβ system is similarly unclear at present.

In our previous study, several observations were made with respect to the clinical relevance of TGFβ isoforms and their receptors in HGSC. Higher levels of *TβRIv1* and *TβRIII* mRNA in post-chemotherapy effusions were associated with a trend for shorter overall survival (OS), with *TβRIII* being an independent prognostic marker in Cox multivariate analysis, suggesting that this pathway mediates aggressive clinical behavior in metastatic HGSC. Analysis of the canonical TGFβ signaling pathway, the R-Smad system, showed reduced levels of Smad2, p-Smad2, and p-Smad3 in effusions compared to those in solid lesions [13].

With the development of proteome analysis technology, MS greatly accelerates signaling research through high-throughput quantification [20].

In this study, we presented quantitative analysis of proteome as a function of deletion of certain TGFβ receptor variants based on examination of two OC cell lines.

Deletion of TβRIIv1 in OVCAR-8 cells resulted in a significant change in protein expression in the KO cells. More than 200 proteins were upregulated in the KO cells. String analysis revealed significant changes in protein pathways labeled as “immune system activation” and as “cytoskeletal organization”. One hundred seventy-five proteins were downregulated. String analysis revealed significant changes in genes involved in protein pathways labeled as “oxidation-reduction processes” and as “cell motility”.

Further examination of ROS levels revealed that the TβRIIv1 OVCAR-8 KO exhibited higher levels of ROS compared to the OVCAR-8 control. This correlates with the proteomic results, showing a downregulation in proteins regulating oxidation-reduction processes, thus leaving the KO cells more susceptible to oxidative stress.

Cellular motility was also examined and, in accordance with the proteomics data, was shown to be significantly lower in both TβRIv1 and TβRIIv1 KOs, showing these receptor variants’ importance in cell migration and disease progression of OC. Further investigation into the effect of the variants revealed that only TβRIv1 reduced the cells’ invasion capabilities. This difference may reflect the fact that these two cell lines are of different histology, as OVCAR-8 is a HGSC cell line and ES-2 originates from a clear cell carcinoma. As clear cell carcinomas are often diagnosed at FIGO stage I and ES-2 cells were indeed isolated from the ovarian tumor, whereas the majority of HGSCs are diagnosed at FIGO stages III–IV, one may speculate that expression differences in these cell lines are also related to disease stage. This may impact the tumor’s ability to metastasize.

Deletion of TβRIv1 in ES-2 cells also resulted in significant changes in protein expression in the KO cells. Fifty-eight proteins were upregulated and 118 proteins were downregulated. String analysis revealed significant downregulation in the “cell death” protein group. This included some proteins involved in the general regulation of cell death and some involved in the negative regulation of cell death. Of the negative-regulating proteins, Western Blot analysis revealed that both TβRIv1 and TβRIIv1 KOs expressed lower levels of pro-MET and MET proteins. MET, otherwise known as Hepatocyte Growth Factor Receptor or c-MET, is known to be upregulated in several types of cancer and to protect from apoptosis [24]. Thus, its downregulation in the receptor variant KOs makes the cells more susceptible to cell death.

EMT a well-known process that occurs in cancer in general, and is known to be regulated by the TGFβ pathway [25]. As part of our objectives was studying the contribution of the splice variants to the progression of OC, we analyzed the expression of regulatory elements of the EMT process, namely, SNAIL and TWIST protein through Western Blot analysis and the lncRNA *MALAT1* through qPCR. Deletion of *TβRIv1* induced a marginal reduction (*p* = 0.06) in TWIST levels, yet had no effect on SNAIL and *MALAT1* expression, while deletion of *TβRIIv1* induced a significant reduction in TWIST expression, along with a significant increase in both SNAIL and *MALAT1* expression. The corresponding upregulation of *MALAT* 1 and SNAIL can be explained through *MALAT1* acting in a ceRNA network, sponging miR-22, and, consequently, releasing *SNAIL* mRNA, allowing it to be translated to protein [26].

In our previous study we showed that the canonical pathway, Smad signaling, is not present in effusion-derived OC samples [13]. Based on clinical significance [19] and correlation to TGFβ receptor levels, we showed in the present study that deletion of the TβRIv1 receptor altered non-canonical signaling in OC cells. JNK phosphorylation depends on the presence of this receptor variant and is shown to be downregulated in the KO. It is well known that JNK has an important role as a death-signaling pathway [27] and its diminished activation may contribute to the downregulation in cell death pathway reported in the proteomics for the same KO.

Combining protein expression changes with the functional assays and gene expression, it can be concluded that the TGF-β receptor variants have specific roles in inducing disease progression. KO of *TβRIv1* effected invasion and downstream non-canonical pathways, whereas KO of *TβRIIv1* effected ROS production and EMT. Both receptor variant KOs contributed to migration and MET protein expressions. These changes indicate the differential roles of these receptor variants on the signaling initiated by TGFβ in OC cells.

## 4. Materials and Methods

### 4.1. Cell Lines

The ES-2 (ATCC^®^ CRL-1978) and OVCAR-8 (CVCL_1629) OC cell lines were obtained from the American Type Culture Collection (ATCC). ES-2 was cultured in DMEM + 10% fetal calf serum (FCS; obtained from Sigma-Aldrich, St. Louis, MO, USA), and OVCAR-8 was cultured in RPMI + 5% FCS. The medium was supplemented with 1% L-glutamine, 1% sodium pyruvate, 1% BME vitamins solution, 1% non-essential amino acids, and 1% Penicillin, Streptomycin, and Amphotericin. All cells were grown in a humidified atmosphere of 95% air and 5% CO2. All reagents were obtained from Biological Industries, Beit-HaEmek, Israel, unless otherwise specified.

### 4.2. CRISPR/Cas9

CRISPR/Cas9 KOs were generated in OVCAR-8 and ES-2 cell lines using the pSpCas9(BB)-2A-Puro (PX459) plasmid #48139 vector, obtained from Addgene (Watertown, MA, USA),and the pSpCas9(BB)-2A-GFP(PX458) plasmid #48138 vector, a kind gift from the lab of Prof. Yehudit Bergman (Hebrew University, Jerusalem, Israel), and processed according to Addgene’s protocol. Briefly, specific, single-guide RNA (sgRNA) inserts (Table 1) were designed to target the introns flanking the exon targeted for deletion (see Figure 1) [15]. Inserts were designed with the help of Zhang Lab scoring [28]. If scoring was insufficient, several sgRNAs were administered in order to increase KO probability. The inserts were 5′ phosphorylated and annealed on a ramp between 25–95 °C. The vector was digested by the BBSI restriction enzyme (New England Biolabs Inc., Ipswich, MA, USA). Extraction and purification of digested plasmid from agarose gel of PCR products was done with Nucleospin^®^ Gel and PCR Clean-up Kit (Macherey-Nagel, Bethlehem, PA, USA). Ligation was performed with T4 DNA ligase (New England Biolabs Inc., Ipswich, MA, USA.). The plasmids were then transformed to competent *DH5α E. Coli* bacteria. Plasmids were extracted with Quick Plasmid Miniprep Kit (#K-3030, Invitrogen, Carlsbad, CA, USA) and sent to sequencing in order to confirm the insertion of the sgRNA. Thereafter, the plasmids were transfected into OC cells with Lipofectamine 3000 (Invitrogen, Carlsbad, CA, USA). In order to ensure transfection, cells were selected by either Puromycin treatment alone (A.G. Scientific Inc., San Diego, CA, USA) or both Puromycin and GFP. Selection was achieved with FACS Aria II (School of Medicine, Hebrew University, Jerusalem, Israel). Sorted cells were then seeded as single-cell colonies, which were then analyzed for KO formation by PCR analysis, where KO formation resulted in expression of the short variant only.

### 4.3. Reverse Transcriptase Polymerase Chain Reaction (RT-PCR)

Total RNA was extracted using the Tri-Reagent kit (Sigma-Aldrich, St. Louis, MO, USA) according to the manufacturer’s protocol. Using the qScript cDNA Synthesis Kit (Quanta Biosciences, Gaithersburg, MD, USA), 500 ng of total RNA was reverse transcribed according to the manufacturer’s protocol. RNA concentration was determined using a Nano-Drop 2000 spectrophotometer (Thermo Fisher Scientific, Waltham, MA, USA).

Then, 500 ng of cDNA were diluted 1:8 with RNase-free water. The cDNA was amplified using Hy-Taq ready mix (Hy-Labs, Rehovot, Israel) according to the manufacturer’s protocol. Products were separated by 1.5% agarose gel. Band density was measured using a computerized image analysis program (Image-J, U. S. National Institutes of Health, Bethesda, MD, USA [29,30]). Then, mRNA expression levels were established by calculating the target mRNA divided by the loading control (*RPLP0*) and compared to the control cell line.

### 4.4. Quantitative Polymerase Chain Reaction (qPCR)

The qPCR detection of each gene was carried out using KAPA SYBR FAST qPCR kit according to the manufacturer’s protocols. PCR specificity was confirmed by appropriate melting curves. The mRNA levels were established by calculating 2^−ΔΔCq^ where ΔΔCq stands for Cq of the loading control *RPLP0* subtracted from Cq of the target gene, following by subtraction of the ΔCq of the control cell line from the ΔCq of the knockout (KO) cell line. Primer sequences are given in Table 2.

### 4.5. Proteomics

Confluent plates of OVCAR-8, ES-2 and KO cells were detached with 1 mM Ethylenediaminetetraacetic acid (EDTA) in PBS. Cell pellets were re-suspended in Urea lysis buffer (Urea 8 M, pH 8, 0.001 M sodium orthovanadate, 0.02 M HEPES, 0.0025 M sodium pyrophosphate, 0.001 M β-glycerophosphate, and 1% protease inhibitor cocktail; Sigma-Aldrich, St. Louis, MO, USA incubated on ice for 20 min, and lysed by gentle sonication (50 Hz; 30 s on, 30 s off; ×3 on ice). Following centrifugation, the supernatant was transferred to a clean tube. Protein concentration was determined by Bradford assay. Cell extracts were run on poly-acrylamide gels and stained with Coomassie Blue to ensure full extraction.

#### 4.5.1. Sample Preparation

The total amount of 50 µg of protein from each sample was subjected to reduction, alkylation, and trypsinization. The samples at a final concentrations of 8 m urea and 100 mm ammonium bicarbonate were reduced with 5 µL of 200 mM dithiotreitol (DTT) for 30 min at 60 °C and alkylated with 20 µL 200 mM iodoacetamide in 100 mm ammonium bicarbonate in the dark for 30 min at room temperature. The excess of iodoacetamide was neutralized by a final addition of 20 µL 200 mM DTT for 30 min in the dark, and the samples were digested in 1 m urea and 25 mm ammonium bicarbonate with modified trypsin (Promega, Madison, WI, USA) at a 1:50 enzyme-to-substrate ratio overnight at 37 °C. An additional second trypsinization was performed for an additional 4 h.

The resulting tryptic peptides were analyzed by liquid chromatography (LC)-MS/MS using a Q exactive-HFX mass spectrometer (Thermo Fisher Scientific, Waltham, MA, USA) fitted with a capillary HPLC. The peptides were loaded onto a C18 trap column (0.3 × 5 mm, LC-Packings) connected on-line to a homemade capillary column (20 cm, 75 micron ID) packed with Reprosil C18-Aqua (Dr Maisch GmbH, Ammerbuch, Germany) in solvent A (0.1% formic acid in water). The peptide mixtures from each treatment were resolved with a linear gradient (5 to 28%) of solvent B (95% acetonitrile with 0.1% formic acid) for 3 h followed by gradients of 5 min (28 to 95%) and 25 min at 95% acetonitrile with 0.1% formic acid in water at flow rates of 0.15 μL/min. Mass spectrometry was performed in a positive mode (*m*/*z* 350–1800, resolution 120,000) using repetitively full MS scan followed by high collision-induced dissociation (at 35 normalized collision energy) of the 15 most dominant ions (>1 charges) selected from the first MS scan.

#### 4.5.2. Proteomic Data Analysis

Data analysis was done by using PD 2.3 Software [31] (Thermo Fisher Scientific, Waltham, MA, USA), and database searches were performed against the Human UniProtKB/TrEMBL database using the Sequest search engine. The mass tolerance for precursor ions and fragment ions was set to 10 ppm and 0.5 Da. Fully tryptic cleavage with two allowed missed cleavages was specified. Carbamidomethyl of cysteine was selected as a fixed modification, and oxidation of methionine was specified as a variable modification. Protein tables were filtered to eliminate the identifications from the reverse database, common contaminants, and single-peptide identifications. The data were quantified by label-free analysis using PD 2.2 Software based on extracted ion currents (XICs) of peptides.

#### 4.5.3. Proteomic Bioinformatics Analysis

Downstream proteomic analyses were done by splitting the results into significantly upregulated (abundance ratio > 2, *p* < 0.05) and significantly downregulated (abundance ratio < 0.5, *p* < 0.05) proteins. Protein lists were uploaded to https://string-db.org/ [accessed on 20 May 2020] to determine protein–protein interactions [32]. Gene Ontology (GO) analysis (http://www.geneontology.org/ [accessed on 20 May 2020] was used for examining biological processes [33,34].

### 4.6. Western Blot Analysis

Samples were lysed with 1% NP-40, 20 mM Tris-HCl (pH 7.5), 137 mM NaCl, 0.5 mM EDTA, 10% glycerol, 1% protease inhibitor cocktail (Sigma-Aldrich, St. Louis, MO, USA), and 0.1% SDS. After centrifugation, the protein content was quantified using the Bradford assay. Then, 25 μg of protein from each sample were resolved by 10% SDS-PAGE (sodium dodecyl sulfate-polyacrylamide gel electrophoresis). The separated extracts were transferred onto Immobilon PVDF membrane (Millipore, Bedford, MA, USA). Following the blocking of nonspecific binding with 5% nonfat milk in TBST, membranes were incubated with the primary antibodies at 4 °C overnight (Erk1/2 #9102, p-Erk1/2 #9106, JNK #9252, p-JNK #9251, AKT #4691, p-AKT #4060, SNAIL #3879, TWIST #46702, MET #3127, and GAPDH #2118 were all purchased from Cell Signaling Technology, Inc. Danvers, MA, USA). Membranes were then incubated with the relevant secondary antibody (Goat anti-Rabbit #111-035-003 and Goat anti-mouse #115-035-003 both), purchased from Jackson ImmunoResearch, West Grove, PA, USA), for 1 h at room temperature (RT). Proteins were detected using an EZ-ECL Chemiluminescence detection kit for HRP (Biological Industries, Beit- HaEmek, Israel). Membranes were imaged with an Image Lab 5.0 gel reader (BioRad laboratories, Hercules, CA, USA). Band density was measured using a computerized image analysis program (Image-J, U. S. National Institutes of Health, Bethesda, MD, USA). Protein expression levels were established by calculating the target protein divided by the loading control (GAPDH) and compared to the calibrator.

### 4.7. ROS Quantification

The 3 × 10^6^-cells/10-mm plate were seeded the day prior to the experiment to approximately 80% confluence. At the start of the experiment, the medium was changed to exclude cells that did not attach. Two hlater, cells were detached using trypsin, and washed twice with warm PBS. Cells were then incubated with 1 µM of The ROS indicator, CM-H_2_DCFDA (#C6827, Invitrogen, Carlsbad, CA, USA), in PBS for 40 min on a rotator at 37 °C. After another wash, cells were re-suspended in 1 mL PBS and incubated for an additional 10 min at 37 °C not on a rotator. Cell suspension was then divided to a 96-black wall, clear-bottom plate, and the fluorescence end point was measured via area scan at an excitation of 480 nm and emission of 520 nm using Cytation 5 Cell imaging Multi-Mode Reader, BioTek, Winsooki, VT, USA.

### 4.8. Scratch Wound-Healing Assay

The day before, 4 × 10^5^ cells/well of OVCAR-8, ES-2, and KO cells were seeded in a six-well plate to create a confluent monolayer. Each well was scratched twice with a sterile tip and was imaged at 0, 6, 24, and 48 h. Wound closure was analyzed by T-scratch software [35,36].

### 4.9. Matrigel Invasion Assay

The 2 × 10^5^ cells of OVCAR-8, ES-2, and KO cells were seeded on filters coated with 25 μg Matrigel, situated in Boyden chambers. A chemoattractant (conditioned medium from the 3T3 fibroblast cell line) was placed on the opposite side. Chambers were then incubated in a CO_2_ incubator for 6 h. The filters were removed, and the presence of invading cells was determined by staining. The invasive cells were quantified by counting the stained cells.

### 4.10. Statistical Analysis

Analysis of RNA expression and metastatic tests was performed using T-test. Significance was determined as *p* < 0.05.

## Figures and Tables

**Table 1:** The sgRNA sequences for the CRISPR/Cas9 method.

Gene	Flanking Region	Forward Sequence (5′-3′)	Reverse Sequence (5′-3′)
*TβRI*	Upstream of Exon 3	GTTGATGTTTATtTCACTCG	CGAGTGAAATAAACATCAAC
	Downstream of Exon 3	GCTTGATGTATATATGACTG	CAGTCATATATACATCAAGC
*TβRII*	Upstream1 of Exon 2	GAAAAAGAGAAACTAGTACC	GGTACTAGTTTCTCTTTTTC
	Upstream2 of Exon 2	TATGGGTGAAAGATCACCAG	CTGGTGATCTTTCACCCATA
	Upstream3 of Exon 2	TGTCTAGTTATAATAATCTT	AAGATTATTATAACTAGACA
	Downstream1 of Exon 2	ATTTAAGACTGGAGAATTTC	GAAATTCTCCAGTCTTAAAT
	Downstream2 of Exon2	GCGATGGCAGTGTACTCATC	GATGAGTACACTGCCATCGC

**Table 2:** Primer sequences.

Gene	Strand	Primer Sequence 5′→3′
*TβRI variant 1*	Sense	TGGACCAGTGTGCTTCGTCTG
	Antisense	CGACCTTTGCCAATGCTTTCT
*TβRI variant 2*	Sense	TCCAACTACTGGTTTACCATTGCTT
	Antisense	CGACCTTTGCCAATGCTTTCT
*TβRII variant 1*	Sense	ACTGCCCATCCACTGAGACAT
	Antisense	TGCACTTTGGAGAAGCAGCAT
*TβRII variant 2*	Sense	CGTTCAGAAGTCGGTTAATAACGA
	Antisense	CACAGGTGGAAAATCTCACATCA
*Malat1*	Sense	GATCCTAGACCAGCATGCC
	Antisense	AAAGGTTACCATAAGTAAGTTCCAGAAAA
*RPLP0*	Sense	CCAACTACTTCCTTAAGATCATCCAACTA
	Antisense	ACATGCGGATCTGCTGCA

## Data Availability

Data available on request.
